# The development of CAR design for tumor CAR-T cell therapy

**DOI:** 10.18632/oncotarget.24179

**Published:** 2018-01-12

**Authors:** Dandan Xu, Guoliang Jin, Dafei Chai, Xiaowan Zhou, Weiyu Gu, Yanyun Chong, Jingyuan Song, Junnian Zheng

**Affiliations:** ^1^ Cancer Institute, Xuzhou Medical University, Xuzhou, Jiangsu, China; ^2^ Center of Clinical Oncology, Affiliated Hospital of Xuzhou Medical University, Xuzhou, Jiangsu, China; ^3^ Jiangsu Center for the Collaboration and Innovation of Cancer Biotherapy, Cancer Institute, Xuzhou Medical University, Xuzhou, Jiangsu, China

**Keywords:** CAR design, antigen target, co-stimulatory molecules, tumor microenvironment, safety

## Abstract

In recent years, the chimeric antigen receptor modified T cells (Chimeric antigen receptor T cells, CAR-T) immunotherapy has developed rapidly, which has been considered the most promising therapy. Efforts to enhance the efficacy of CAR-based anti-tumor therapy have been made, such as the improvement of structures of CAR-T cells, including the development of extracellular antigen recognition receptors, intracellular co-stimulatory molecules and the combination application of CARs and synthetic small molecules. In addition, effects on the function of the CAR-T cells that the space distance between the antigen binding domains and tumor targets and the length of the spacer domains have are also being investigated. Given the fast-moving nature of this field, it is necessary to make a summary of the development of CAR-T cells. In this review, we mainly focus on the present design strategies of CAR-T cells with the hope that they can provide insights to increase the anti-tumor efficacy and safety.

## INTRODUCTION

**Figure 1:**
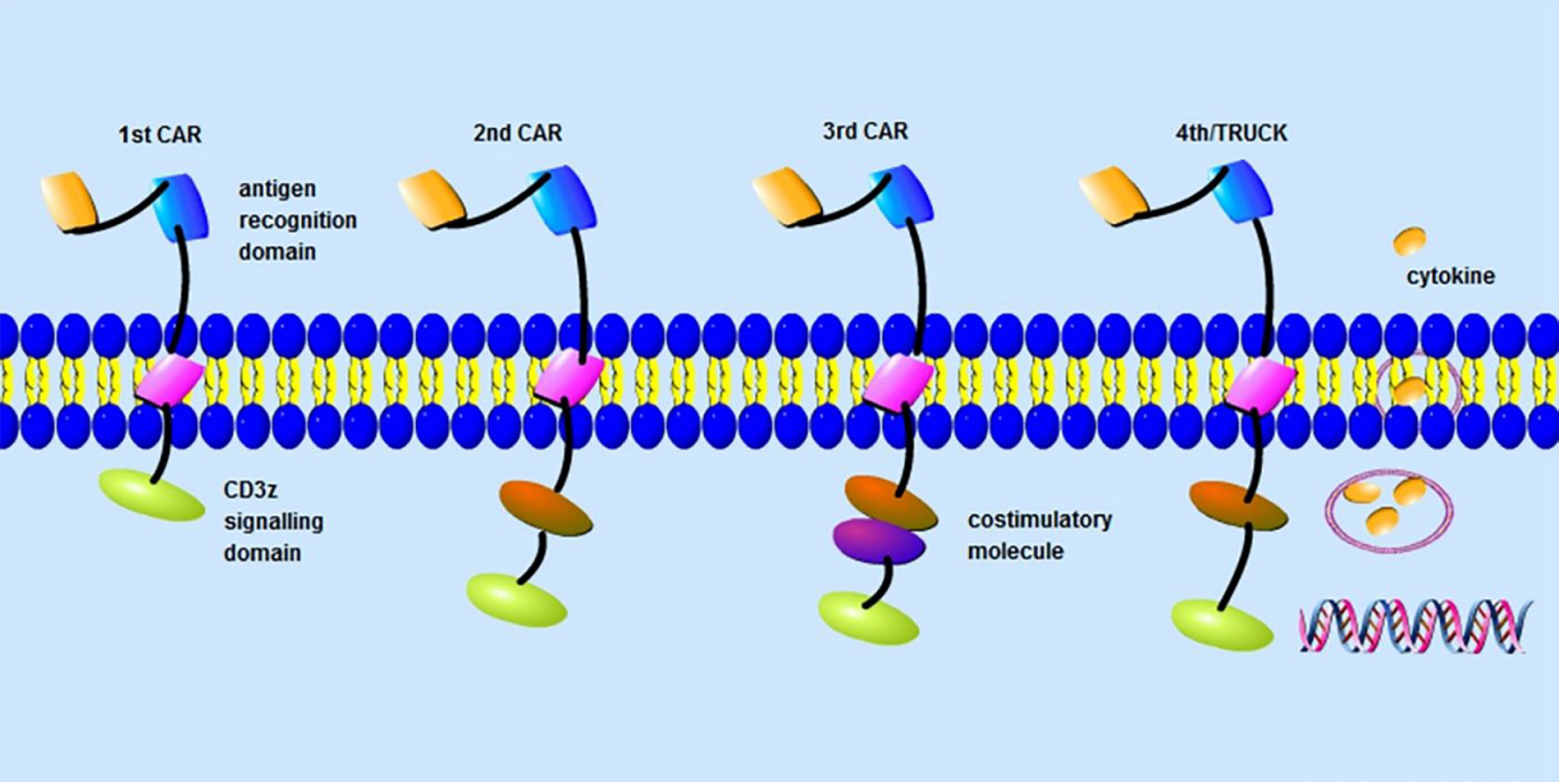
CAR-T-cell design Chimeric antigen receptors (CARs) are composed of an extracellular domain, a transmembrane domain and an intracellular signaling domain. First generation CARs only have a CD3z signalling domain. By contrast, second generation CARs have a costimulatory signalling domain to enhance the signal function of the CD3z signalling domain. In third generation CARs, two costimulatory signalling domains are added to amplify anti-tumor effect of secondgeneration CARs. While in the fourth generation CARs (TRUCKs), cytokine genes are added.

Today the risk to develop cancer is quite high and the number of tumor patients unfortunately is still even increasing [1, 2]. If not slowed down, it is expected that the global annual new cases will reach 15 million by 2020 [3]. As a form of cancer treatment, the emergence of chimeric antigen receptors (CARs) T cell therapy brings hope to tumor patients. The CAR-T cells can target tumor antigens independent of MHC restriction [4, 5], which include an extracellular antigen binding domain, a trans-membrane portion and an intracellular signalling domain (Figure 1) that is very important to the complete activation of CAR-T cells [5, 6]. Upon the recognition of specific antigens, CAR-T cells are activated to proliferate and secrete cytokines. CAR-T cells can promote cancer killing and has shown promise for the immunotherapy of some human malignancies [7, 8]. But, the treatment efficacy of solid tumors utilizing CAR-T cells is unsatisfactory and a set of challenges still are not solved, such as antigen specificity, mechanisms of exhaustion and safety issues [9, 10].

## EXTRACELLULAR ANTIGENIC TARGETS

The identification of targets happens before and is a prerequisite of CAR-T cell therapy. In order to avoid damage to healthy tissues caused by CAR-T cell therapy, the targets must be restricted on tumor cells [11, 12]. So far, a variety of tumor associated antigens (TAAs) have been targeted to achieve ideal therapeutic effect [13, 14].

### Hematological malignancies

**Figure 2:**
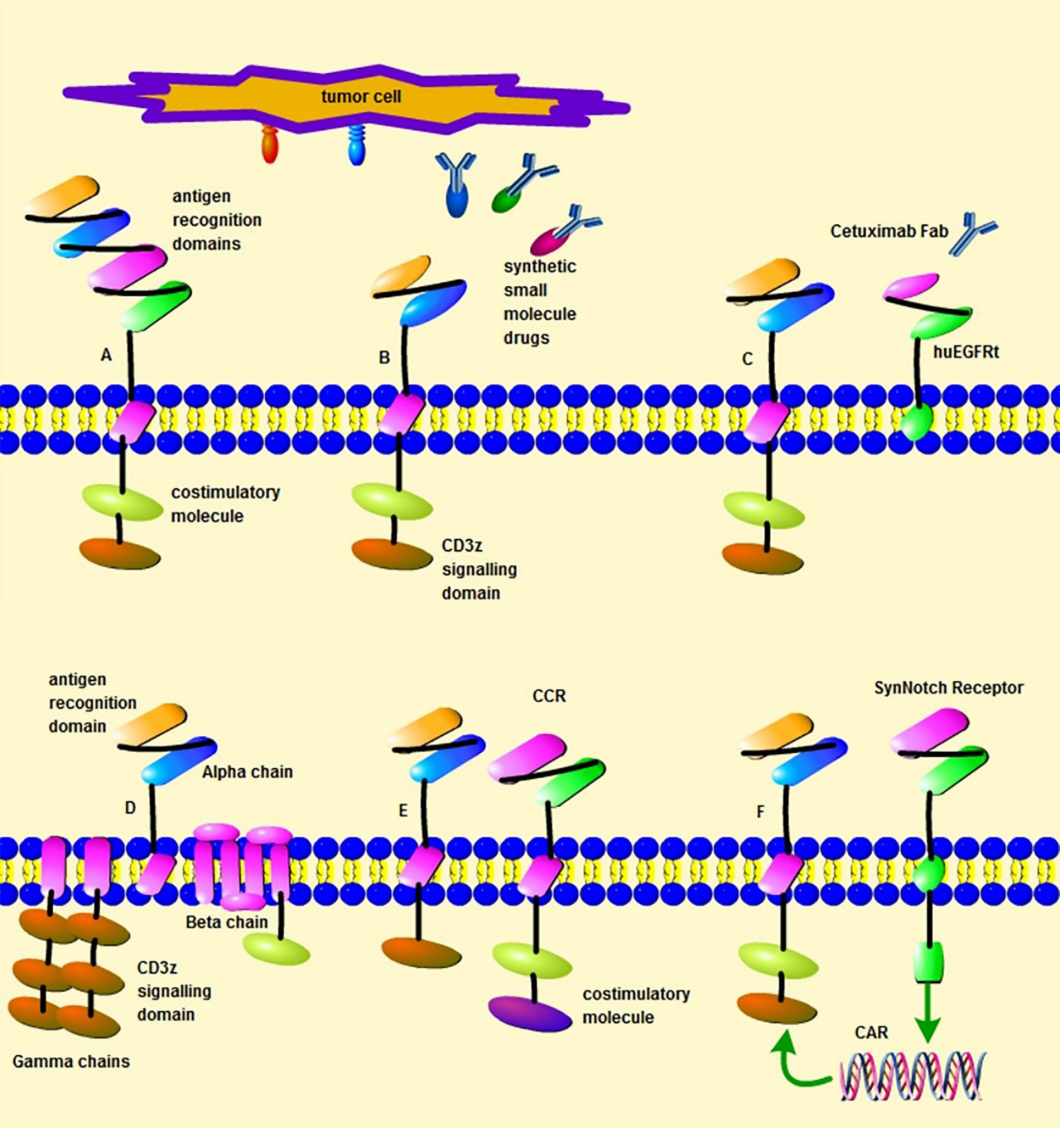
Different design strategies of CAR T cells (**A**) A bi-specific CAR targeting two different antigens. (**B**) A CAR that target tumor antigens through synthetic small molecule drugs, such as the avidin-CAR, sCAR or UniCAR. (**C**) A CAR designed with huEGFRt. (**D**) The design of multi-chain CAR based on FcεRI receptor scaffold (**E**) A suboptimal CAR and a chimeric co-stimulatory receptor (CCR) expressed by one T cell. (**F**) The expression of a CAR induced by a synNotch receptor within one T cell.

Within the past few years, clinical trials of CAR-T cell therapy were tested in hematological malignancies. For example, CD19-targeted CARs to treat B cell cancers are up to 70%–90% response rate in acute and chronic leukemias [15–18]. Although the great success with CD19 specific CARs, CD19 escape variants have been confirmed after therapy and responding patients with subsequent target loss have a recurrence of the disease [19–21]. To overcome such antigen target issues about escape variants, one method is to investigate other tumor antigen targets, such as CD22 [22], CD20 [23], CD138 [24], CD33 [25], CD123 [26], inactive tyrosine-protein kinase transmembrane receptor ROR1 (ROR1) [27], immunoglobulin kappa chain (Igκ) [28], B-cell maturation antigen (BCMA) [29], and Lewis Y antigen (LeY) [30, 31] (Table 1). Another way is to develop new strategies to design CAR-T cells, such as bi-specific chimeric antigen receptors. For example, the design of CD19/CD20 tandem CAR-T cells that can kill tumor cells efficiently when encountering either of the antigens [32, 33]. In addition to the methods above, the concepts of double CARs or dual receptors within one T cell, switchable CARs mentioned below also can be used here to prevent the development of antigen escape variants (Figure 2). Besides CD19 escape variants, the missing of HVEM (Herpes Virus Entry Mediator) also is reported, which caused lymphomas *in vivo* due to the destruction of inhibitory interactions between the HVEM and BTLA (B and T lymphocyte attenuator) receptors. CAR-T cells that secrete HVEM have shown great therapeutic efficacy against xenografted lymphomas *in vivo* [34].

**Table 1:** CAR-T-cell targets for the treatment of hematological tumors

Target	CAR structure	Malignancy	Reference
BCMA	CD3ζ and 41BB	MM	NCT02215967 [29]
CD19	CD3ζ and CD28; CD3ζ and 41BB KIR2DS2 and DAP12-	Lymphoma; Leukemia	NCT01044069 [17] NCT01626495 [18] NCT02685670 [105] [114]
CD22	CD3ζ and CD28	FL; NHL; DLBCL; ALL	NCT02315612 [22]
CD20	CD3ζ; CD3ζ and 41BB-	CD20positive malignancies	NCT01735604 [23]
CD138	CD3ζ and 41BB	MM	NCT01886976 [24]
CD33	CD3ζ and 41BB	AML	NCT01864902 [25]
CD123	CD3ζ and CD28	AML	NCT02159495 [26]
CD19 CD20	CD3ζ and 41BB	Leukemia; Lymphoma	NCT03097770 [32]
CD19 PSMA	CD3ζ and CD28 PD-1 or CTLA4	Leukemias	[67]
FITC-CD19 Ab	CD3ζ and CD28	CD19 positive cancers	[126]
Igκ	CD3ζ and CD28	CLL	NCT00881920 [28]
LeY	CD3ζ and CD28	AML	NCT01716364 [30]
ROR1	CD3ζ and 41BB	CLL; SLL	NCT02194374 [27]

AML, acute myeloid leukaemia; ALL, acute lymphoblastic leukaemia; BCMA, Bcell maturation antigen; CLL, chronic lymphocytic leukaemia; CTLA4, Cytotoxic T lymphocyte associated antigen 4; DLBCL, diffuse large Bcell lymphoma; DAP12, DNAX-activating protein of 12 kDa; FL, follicular lymphoma; FITC, fluoresceine isothiocyanate; Igκ, immunoglobulin kappa chain; KIR2DS2, stimulatory killer immunoglobulin-like receptor 2DS2; LeY, Lewis Y antigen; MM, multiple myeloma; NHL, nonHodgkin lymphoma; PSMA, prostatespecific membrane antigen; PSMA-CAR (iCAR) [67], inhibitory chimeric antigen receptor; PD-1, programmed death 1; ROR1, inactive tyrosineprotein kinase transmembrane receptor ROR1; SLL, small lymphocytic lymphoma.

### Solid tumors

With the comforting results of CD19 CARs, much attention has been paid to the development of CARs to look for effective methods to treat solid tumors successfully. About solid tumors, many tumor-associated antigens are also targeted to get the optimal efficacy (Table 2).

**Table 2:** CAR-T-cell targets for the treatment of solid tumors

Target	CAR structure	Malignancy	Reference
Biotin	CD3ζ, CD28 and 41BB	EGFRvIII positive cancer	[125]
CD171	CD3ζ and 4-1BB; CD3ζ, CD28 and 4-1BB	Neuroblastoma	NCT02311621 [70]
EGFRvIII	CD3ζ and 41BB CD3ζ and ICOS-	Glioma	NCT02209376 [40] [107]
FAP	CD3ζ and CD28 KIR2DS2 and DAP12-	Mesothelioma; Lung cancer	[114]
FR	CD3ζ and CD27	Ovarian cancer; Breast cancer	[98]
Glypican-3	CD3ζ, CD28 and 41BB	Hepatocellular carcinoma	NCT02395250 [112]
HER2	CD3ζ and CD28	HER2 positive cancer; Sarcoma	NCT02713984 [61] NCT00902044 [60]
HER2 MUC1	CD3ζ and CD28	Breast cancer	[62]
HER2 IL13Rα2	CD3ζ and CD28	Glioblastoma	[47]
IL13Rα2	CD3ζ; CD3ζ and 41BB CD3ζ and CD28 CD3ζ, CD28 and 41BB CD3ζ, CD28 and OX40-	Glioma	NCT02208362 [45] [46]
Mesothelin	CD3ζ; CD3ζ and CD28 CD3ζand 41BB CD3ζ and ICOS KIR2DS2 and DAP12-	Mesothelioma; Pancreatic cancer; Non-small cell lung cancer	NCT01355965 [49] NCT02465983 [50] [106] [114]
Mesothelin CD19	CD3ζand 41BB	Pancreatic cancer	NCT02465983[50]
MUC1	CD3ζ and 41BB	MUC1 positive solid tumor	NCT02587689 [54]
NKG2D	CD3ζ; CD3ζ and DAP10 CD3ζ and 41BB CD3ζ and CD28	Ovarian cancer Ewing sarcoma	[71, 72]
PSMA	CD3ζ and CD28	Prostate cancer	NCT01140373 [64] NCT00664196 [65]
PD1 and CD19; PD1 and Mesothelin;	CD3ζ and CD28 CD3ζ, CD28 and 41BB	PD-L1 positive cells	[87]

DAP12, DNAX-activating protein of 12 kDa; DAP10, DNAX-activating protein of 10 kDa; EGFRVIII, epidermal growth factor receptor variant III; FAP, fibroblast activation protein; FR, folate receptor; FL, follicular lymphoma; HER2, human epidermal growth factor receptor 2; IL13Rα2, interleukin 13 receptor α2; KIR2DS2, stimulatory killer immunoglobulin-like receptor 2DS2; MUC1, mucin 1; NKG2D, Natural Killer Group 2D; PSMA, prostatespecific membrane antigen; PD-1, programmed death 1; PD-L1, programmed death ligand 1.

Epidermal growth factor receptor (EGFR) and EGFR variant (EGFRvIII) are over-expressed in many cancer types and are commonly associated with the malignancy of glioblastoma [35, 36]. The expression of EGFRvIII within a cell is often associated with survival, invasion, angiogenesis and resistance against radiation and chemotherapy [37, 38]. EGFRvIII specific CAR-T cells have shown great antitumor efficacy in preclinical studies and now they have been being evaluated in clinical trials [39, 40]. EGFR also could be modified as a useful tool, which retains a cetuximab binding site and lacks domains I and II and its cytoplasmic tail. Cetuximab can recognize the truncated EGFR (huEGFRt) so that CAR-T cells expressing the truncated EGFR can be selected, tracked and ablated *in vivo* after administration of cetuximab [41].

Interleukin 13 receptor α2 (IL13Rα2) is a glioma-associated antigen and also is associated with a reduced survival rate of patients [42, 43]. In a study, after CAR-T cell treatment, regression of tumors along with corresponding increases of cytokines and immune cells was observed [44, 45]. However, the IL13Rα2 specific CARs can also recognize interleukin 13 receptor α1 (IL13Rα1). To solve the problem, IL13Rα2 specific single-chain variable fragment (scFv) 47 is used as an antigen binding domain and the specificity indeed has been enhanced [46]. Specificity could also be improved by a CAR targeting two or more antigens. IL13Rα2 and human epidermal growth factor receptor-2 (HER2) specific CARs are designed with CD3z and CD28 domains to make tandem CARs (TanCARs) [47]. These CAR-T cells can distinctively and effectively recognize tumors, mitigate antigen escape and have also shown enhanced persistence in the presence of the both targets.

Mesothelin is a TAA expressed by many malignant cancers [48]. CARs specific for mesothelin have been investigated in clinical trials to treat patients with pancreatic cancer and malignant pleural mesothelioma [49, 50]. In the terms of persistence, patients with pancreatic cancer have been treated with T cells that simultaneously express two CARs targeting mesothelin and CD19 in clinical trials [50]. Thus, the influence of antibodies on the mesothelin directed CARs can be blocked due to the eradication of B cells by the CD19-specific CARs and then these CAR-T cells can function for a long time.

Aberrant expression of glycoform has been found on the cell membrane mucin-1 (MUC1), a large protein carrying O-glycan over-expressed by most adenocarcinomas [51, 52]. CARs targeting the MUC1 glycopeptide epitope are designed based on a monoclonal antibody (5E5) and these CAR-T cells have shown ability to eliminate pancreatic tumors [53–55]. Interleukin-4 (IL-4) has several pathophysiologic and therapeutic links to cancers and can promote the function of CAR-T cells. MUC1 specific CAR-T cells engineered with IL-4 receptor ectodomain have shown enhanced resistance to immunosuppressive cytokines and improved anti-tumor efficacy [56, 57].

HER2 is a member of receptor tyrosine-protein kinase family, which is over-expressed by many tumor cells and also is expressed by some epithelial cells [58, 59]. In clinical trials, patients with tumors expressing HER2 have been treated with second generation HER2 targeted CARs (CD28/CD3z) [60, 61]. Several research groups are attempting to design two CARs in a single T cell, which can specially recognize tumor cells. In a trial, HER2 and MUC1 specific CARs with CD3z and co-stimulatory molecule respectively within one T cell have been designed, which can eliminate tumor cells efficiently and offset tumor antigen escape variants when encountering target cells co-expressing MUC1 and HER2 [62].

Most prostate-cancer cells and many tumor-associated neo-vasculatures express prostate specific membrane antigen (PSMA) [63, 64]. Thus anti-angiogenic effects together with direct anti-tumor effects might be able to get by PSMA specific CARs [65]. To improve the specificity of CAR-T cells, PSMA expressed by normal tissues is targeted to provide negative signaling to the PSMA specific dual targets CAR-T cells with the co-stimulatory molecule of programmed death-1 (PD1) or cytotoxic T lymphocyte associated antigen 4 (CTLA4) [66, 67]. The strategy of inhibitory chimeric antigen receptors (iCARs) above can be used not only to enhance antigen recognition, but also to increase safety.

Neural cell adhesion molecule L1, also named CD171, is expressed on many tumors, but also on normal tissues [68]. However, the expression pattern of CD171 by cancers is glycosylated, different from that of normal cells. CARs have been developed to target the glycosylated CD171 expressed on malignant cells and these T cells have been demonstrated safety without on-target off-tumor toxicity [69]. Moreover, clinical trials using CD171 specific CARs are also in progress [70]. In addition to targeting tumor antigens, receptors or ligands can also be targeted to enhance specificity of CARs, such as the use of Natural Killer Group 2D (NKG2D) [71, 72].

### Tumor microenvironment

### Infiltration and trafficking

T cells must be able to traffic to tumor sites in order to exert their effector functions *in vivo*. Extracellular matrix (ECM) is the main barrier of transport, which contains the heparan sulfate proteoglycans (HSPGs), the main component of ECM [73]. T cells must get rid of obstacle of HSPGs in stroma-rich tumor microenvironment to reach tumor sites. However, T cells have no ability to express the enzyme heparanase (HPSE) to degrade heparan sulfate proteoglycans. Therefore, CAR-T cells that can secrete heparanase are engineered, which can promote infiltration and anti-tumor activity [74]. Chemokine receptors can also be used to enhance traffic. For example, CD30-directed CAR-T cells engineered with CC-chemokine receptor 4 (CCR4) have enhanced migratory capacity in murine Hodgkin’s lymphoma xenograft models [75]. CAR-T cells expressing CC chemokine receptor 2b (CCR2b) also have improved migration in mesothelioma and neuroblastoma that naturally secrete large quantities of CC chemokine ligand 2 (CCL2) [76, 77].

### Target tumor vasculatures

Abundant blood vessels in tumor tissues can express immunosuppressive molecules and promote the growth of tumors. Thus, targeting tumor vasculature is a strategy to improve cell immunotherapy with CARs. Studies have confirmed that poor prognosis and metastasis of tumors is due to the over-expression of vascular endothelial growth factor (VEGF) and their receptors (VEGFR) in the tumor microenvironment [78]. Now vascular endothelial growth factor receptor-2 (VEGFR2) has been targeted to treat patients with metastatic tumors and enhanced efficacy has been achieved by these T cells [79]. Anti-angiogenic therapy also can lead to increased expression of adhesion molecules and chemokines that can enhance infiltration [80].

### Target immunosuppressive cells and factors

At present, the main targets of CAR-T cell therapy for tumors are PSMA, Mesothelin, HER2, EGFR, and so on. To date, it lacks methods to overcome the inhibitory effect of tumor microenvironment on CAR-T cells. Therefore, novel CARs need to be developed to improve inhibition of tumor microenvironment and enhance anti-tumor abilities. It has been pointed that PD1 or CTLA4 can inhibit the function of T cells in the tumor microenvironment [81, 82]. However, checkpoint inhibiting antibodies can block the inhibitory signal to the T cells and have achieved successful results for the treatment of different tumor types [83–86]. Now, CARs that can secrete PD1 and/or CTLA4 antibodies have been designed to improve immunosuppression and enhance anti-tumor effect in clinical trials and it has been demonstrated that tumor volume can be decreased by PD1 specific CARs [87–89]. Our research group is designing CTLA4-specific CAR-T cells to study the effects of improving immunosuppressive microenvironment and enhancing anti-tumor cytotoxicity. Adenosine, as a potent immunosuppressive factor, is regarded as a potential target [90]. It is reported that adenosine A2A receptors (A2ARs) can be up-regulated by CAR-T cells to exert a negative immune reaction when combining adenosine. Blockade of A2ARs has achieved great responses significantly, particularly in the help of PD-1 blockade [91]. Thus, new CARs may be designed to secrete adenosine antagonists or adenosine antagonists and PD1 antibodies to enhance anti-tumor efficacy.

## SPATIAL DISTANCE AND SPACER REGION

Some studies have reported that the distance between antigen recognition domains and the specific antigen targets can affect the function of CARs. It has been demonstrated that the epitope near a more-proximal position on the membrane can activate CAR-T cells more efficiently [92–94]. In a trial, CARs can greatly recognize and attack tumor cells *in vitro* through targeting an epitope in a distal position on the membrane with a shortened extracellular spacer region compared with a longer one [46, 95]. It is a question whether the CAR-T cells without a hinge domain can enhance tumor killing. Therefore, two kinds of CARs, with or without a hinge domain, have been designed to study the problem. Finally, it is concluded that a hinge can enhance the expansion and anti-tumor efficacy for some specific CAR-T cells [96, 97]. Thus, the antigen target location and hinge length should be taken into account when designing CARs, which are critical for the activity of CAR-T cells.

## INTRACELLULAR SIGNALLING DOMAINS

Many co-stimulatory molecules have been investigated, including CD28, 4-1BB (CD137), CD27 and OX40 (CD134), which have been incorporated into CARs to further enhance therapeutic effect [98, 99] (Table 2). With the development of co-stimulatory molecules, CAR-T cells have experienced four generations of development (Figure 1). The first generation only utilizes CD3z chain typically to provide an activation signal. Early studies show that the persistence of the first generation CARs is superior, but the expansion ability and anti-tumor efficacy are unsatisfactory [99]. Subsequently, a co-stimulatory molecule is added into the structures of CARs to augment the proliferation and responses, which is so-called second generation CARs [100]. It has been pointed that CD28 can enhance the telomere length, which can affect the persistence and anti-tumor efficacy of T cells [101, 102]. Thus, senescent T cells can be regenerated by restoration of CD28 expression [103]. CAR-T cells with CD28 or 4-1BB signaling domain have shown potent anti-tumor efficacy *in vivo* for B cell malignancies [104, 105]. ICOS co-stimulatory domain also has been used and CARs with ICOS tend to have enhanced survival time than CARs with CD28 or 4-1BB co-stimulatory domain [106, 107]. In order to further strengthen the function of the second generation CARs, the third generation has been designed that has two co-stimulatory molecules [108]. CAR-T cells with CD28 and 4-1BB domains have shown enhanced functionality and increased persistence [109–112]. In addition to these co-stimulatory molecules mentioned above, some other molecules are also being studied, such as CTLA-4 or PD-1. Antigen specific suppression of CAR-T cells with CTLA-4 or PD-1 can be achieved to prevent the damage of inadequate T cell specificity to normal tissues [67, 113]. Stimulatory killer immunoglobulin-like receptor (KIR) KIR2DS2 and DNAX-activating protein of 12 kDa (DAP12) also are used to replace CD3z and co-stimulatory molecule to enhance the proliferation and function of CAR-T cells, which can destroy immunotherapy-resistant solid tumors efficiently [114]. Different from the first three generation CARs, cytokine genes that can improve the activation and expansion of T cells and promote the resistance to immunosuppression have been introduced into the fourth generation CARs (TRUCKs) and these CAR-T cells modified with cytokine genes can use some valid components of the tumor microenvironment to amplify anti-tumor efficacy [115–117].

## SAFETY

Although remarkable clinical efficacy, it is still difficult to apply the current CAR-T therapy generally due to the restriction of serious treatment-related toxicities [118]. Bi-specific chimeric antigen receptors and dual CARs strategies mentioned above can help to reduce the risk of development of side effects. Besides, dual receptors in one T cell also can prevent the development of side effects and increase specificity. For instance, a CAR and a chimeric co-stimulatory receptor (CCR) or a CAR and a synthetic Notch receptor are designed in one T cell to target two different antigens. These T cells only eliminate tumors with both antigens but do not destroy cells with either antigen alone [119, 120]. Multi-chain CARs based on FcεRI receptor scaffold also have been investigated to increase safety. FcεRI receptor scaffold has three different polypeptide chains (alpha, beta and gamma) and these polypeptide chains are substituted by an antigen recognition domain, a co-stimulatory molecule and CD3z respectively. Between the antigen binding domain and a hinge domain, FKBP domains and/or FRB are incorporated, which have a high affinity to the rapamycin and FKBP-rapamycin complex respectively. With the application of a small molecule of rapamycin or analog of rapamycin, antitumor cytotoxicity and advantage for safety are shown by these designed CAR-T cells [121]. A split-receptor design has been used to engineer CAR-T cells, which have antigen binding and intracellular signaling domains on separate polypeptides. These T cells can be activated only on the presence of the heterodimerizing small molecule and tumor antigens. Moreover, the activity of these T cells is titratable by the dose of the small molecules, which increase the safety of CAR-T cells application [122] (Figure 2).

It is highly desirable to design universal CARs that have the ability to recognize multiple TAAs and minimize the risk of treatment-related toxicities. A study reported a novel and universal CAR strategy that can extend the specificity and safety potential of CAR-T cells by using a biotin-avidin system [123, 124]. EGFRvIII+ gliomas were targeted by biotinylated monoclonal antibody (biotin-4G1) and then avidin-CARs were used against the biotin-4G1. This therapeutic strategy is proved valid by EGFRvIII+ glioma-bearing mice [125]. Other analogous “switch” molecules also have been explored to regulate CAR-T cells activity *in vivo* to minimize toxicities, while maintaining potent anti-tumor activity, such as the switch molecules modified with fluoresceine isothiocyanate (FITC) or peptide neo-epitopes (PNE). FITC or PNE -specific CAR-T cells kill tumors dependent on the presence of switch molecule, which can enhance the activity of the CAR-T cells by dose titration [126–129]. To reduce the risk of side effects and broaden the range of application of CAR-T cells, a modular CAR platform (UniCAR) was developed to target different tumor antigens through different specific targeting modules (TMs) that have incorporated a peptide epitope E5B9. The function of E5B9 specific CAR-T cells is completely dependent on the presence of specific TMs and specific targets. Moreover, the activity of these cells can be turned on and off by the TMs [130–132]. However, it is a problem whether these synthetic small molecules are absolutely safe after long-term application. We need to think about the problems that whether similar elements to these molecules will be produced in the body and whether the body will be resistance to these molecules.

The development of gene editing technology has also helped to improve safety. The activity of CAR-T cells can be eliminated by activating the suicide gene caspase-9 (iCasp9) that can effectively induce apoptosis of CAR-T cells to overcome side effects [133]. Another elimination gene is the truncated EGFR mentioned above. The activity of CAR-T cells can be rapidly eliminated with administration of cetuximab to prevent the events of serious toxicities [41]. However, suicide gene strategies can result in terminating therapeutic responses because of eliminating T cells indiscriminately. Moreover, gene editing also is capable of producing CAR-T cells that have ability to avoid graft versus host disease (GvHD) induced by allogeneic CAR-T cells through eliminating the expression of the endogenous T cell receptor (TCR) to enhance safety [134, 135].

## CONCLUSIONS

In recent years, CAR-T cell immunotherapy has achieved highly effective results in treating hematological malignancies and achieved much progress on the aspects of antigen targets, intracellular signal domains and the combined application of immune cells and synthetic small molecule drugs. Despite significant progress, some major challenges still have not been solved in engineered T cells to treat solid tumors and have remain significant barriers to its broader clinical application, especially in terms of specificity, persistence, safety, and immunosuppressive microenvironment [136]. We expect the reliable, safe, and effective CAR-T cells and extend it toward the treatment of a broad range of tumors in the future.
